# Emergency Cranial Neurosurgery: An Audit of Operative Burden in a Specialized Neurosurgical Center in a Resource-Limited Setting

**DOI:** 10.7759/cureus.81668

**Published:** 2025-04-03

**Authors:** Shehzad Safdar, Usama Mansoor, Noor U Ain, Mubashir Malik, Sana Saleem, Shahzeb Ahmad

**Affiliations:** 1 Neurosurgery, Beaumont Hospital, Dublin, IRL; 2 Neurosurgery, Punjab Institute of Neurosciences, Lahore, PAK

**Keywords:** burden, cranial neurosurgery, emergency, hydrocephalus, low middle income country, pakistan, specialized center, trauma

## Abstract

Background

Neurosurgical emergencies pose significant challenges not only to healthcare systems but also to society and the economy. This audit examines the burden of emergency cranial neurosurgery procedures at a specialized center, highlighting the volume of cases and underscoring the need for additional dedicated neurosurgical facilities to meet the growing demand.

Objective

To assess the burden of emergency cranial neurosurgery procedures at the Punjab Institute of Neurosciences (PINS) in Lahore, Pakistan.

Methodology

This retrospective observational audit was conducted at the PINS, Lahore, analyzing data from patients who underwent emergency cranial neurosurgery over six months. A consecutive sampling technique was used, including all eligible patients while excluding those with incomplete records or non-cranial procedures. Data were collected from various hospital records and cross-verified for accuracy. Patients who underwent multiple procedures in a single surgery were recorded as a single entry. Statistical analysis was performed using IBM SPSS Statistics for Windows, Version 27.0 (Released 2020; IBM Corp., Armonk, NY, USA).

Results

A total of 562 patients, aged 7-90 years, with a mean age of 38.18 ± 18.42 years, underwent emergency cranial neurosurgery. Males accounted for 415 cases (73.84%), resulting in a female-to-male ratio of approximately 1:2.82. Traumatic cases comprised 241 patients (42.88%), with extradural hematoma diagnosed in 110 patients (34.27% of traumatic cases; 19.57% of total cases), acute subdural hematoma in 94 patients (29.28% of traumatic cases; 16.73% of total cases), and hemorrhagic contusion in 34 patients (10.59% of traumatic cases; 6.05% of total cases). Nontraumatic cases accounted for 321 patients (57.12%), with non-tumor hydrocephalus observed in 70 patients (29.05% of nontraumatic cases; 12.46% of total cases), intracerebral hematoma in 37 patients (15.35% of nontraumatic cases; 6.58% of total cases), and shunt malfunction in 23 patients (9.54% of nontraumatic cases; 4.09% of total cases). The most common surgical interventions performed were craniotomy for extradural hematoma in 106 patients (18.86%), ventriculoperitoneal shunt placement in 79 patients (14.06%), and tracheostomy in 54 patients (9.61%).

Conclusions

This audit highlights trauma and hydrocephalus as the leading causes of emergency cranial neurosurgical procedures. In developing countries like Pakistan, specialized neurosurgical centers, particularly their emergency departments and operating theaters, face a substantial patient load, emphasizing the urgent need for expanded facilities and resources.

## Introduction

Neurosurgical emergencies encompass a wide range of traumatic and nontraumatic conditions, requiring a comprehensive approach to management. Traumatic injuries, often resulting from vehicular accidents, falls, sports-related incidents, assaults, and natural disasters, pose a significant global burden, with preventable incidents contributing to increasing incidence rates. Beyond affecting individual patients, these injuries also have far-reaching consequences for families, society, and healthcare systems [1,2]. Traumatic brain injuries (TBIs) and spinal cord injuries (SCIs) represent a major proportion of neurotrauma cases worldwide, with approximately 27.08 million new TBIs and 0.93 million SCIs reported annually [3,4]. A review of post-traumatic headaches found that 50% of TBI patients across nine studies experienced this complication, significantly reducing their quality of life [5]. Similarly, a systematic review revealed that 43.2% of pediatric-onset SCI patients had complete injuries, with anxiety and depression being common long-term outcomes [6]. In Pakistan, the estimated incidence rate of neurotrauma is 81 per 100,000, with a 15% mortality rate and surgical intervention required in 20-23% of cases [4,7].

Nontraumatic neurosurgical emergencies, including hydrocephalus, spontaneous intracerebral hemorrhage (ICH), and chronic subdural hematoma, further strain healthcare systems. Hydrocephalus has a prevalence of approximately 85 per 100,000 individuals, while ICH affects 15-19 per 100,000 annually, and chronic subdural hematomas occur in 17-40 per 100,000 cases [8-11]. Managing these emergencies involves a combination of outpatient care, surgical interventions, pharmacotherapy, multidisciplinary collaboration, rehabilitation services, and hospital admissions. While advances in therapeutic modalities have improved survival rates, disability remains a significant concern. In resource-limited settings, these challenges are exacerbated by a shortage of specialized neurosurgical centers, further increasing the strain on existing facilities handling emergency cases [12-14].

Addressing these issues requires a concerted effort in prevention, early intervention, and the strengthening of healthcare infrastructure to mitigate the societal and economic impact of neurosurgical emergencies. This audit aims to quantify the burden of emergency cranial neurosurgery procedures at a specialized neurosurgical center. Assessing this burden will highlight the high volume of emergency neurosurgical cases and underscore the urgent need for additional dedicated centers to meet the growing demand.

## Materials and methods

Following approval from the hospital’s ethical review committee (approval number 1873/IRB/PINS/Approval/2024), this audit was conducted using a retrospective observational design. The study took place at the Punjab Institute of Neurosciences (PINS) in Lahore over six months, from January 1, 2022, to June 30, 2022. As Pakistan’s leading facility for neurosurgical, neurological, and neuroradiological care, PINS operates two emergency theaters around the clock. However, the overwhelming number of referred patients places considerable strain on its resources. Hospital records indicate that the most frequently performed emergency surgeries involve trauma management, hydrocephalus treatment, ICH evacuation, chronic subdural hematoma drainage, and tracheostomy procedures. The findings of this audit are expected to provide crucial data for healthcare policymakers and planners, emphasizing the urgent need to expand neurosurgical capacity to better meet the population’s critical needs.

A non-probability, non-randomized consecutive sampling technique was employed. The sample included all patients who underwent surgical intervention in the emergency neurosurgery operation theaters at PINS during the study period, totaling 562 patients.

Inclusion criteria

Patients were eligible for inclusion if they underwent surgical intervention in the emergency neurosurgery operation theaters of PINS during the study period.

Exclusion criteria

Patients were excluded if their records were incomplete for any variables necessary for analysis. Additionally, those who underwent surgeries for spinal procedures, lumbar puncture, or central vein catheter placement were not included in the study.

Data collection

Data were extracted from hospital admission records, registers maintained by emergency operation theater surgeons, anesthetists, and nurses, as well as patient files and operation notes. Records with missing essential demographic or procedural details were excluded. If a patient had multiple lesions or injuries addressed in a single surgical session, the case was recorded as a single entry to streamline data analysis. To ensure accuracy, a random sample comprising 10% of the records was cross-checked by two independent researchers.

For statistical analysis, IBM SPSS Statistics for Windows, Version 27.0 (Released 2020; IBM Corp., Armonk, NY, USA) was used. Quantitative variables, such as age, were presented as mean and SD, while categorical variables, including gender, case type (traumatic/nontraumatic), and surgical procedures, were reported using frequency and percentage.

## Results

During the study period, 562 patients, ranging in age from 7 to 90 years, were analyzed. The mean age was 38.18 years (±18.42), with 45 years being the most frequently occurring age. Of these patients, 147 were female (26.15%), while 415 were male (73.84%), resulting in a female-to-male ratio of approximately 1:2.82.

The analysis showed that non-traumatic cases (321 patients, 57.12%) slightly outnumbered traumatic cases (241 patients, 42.88%), with a ratio of approximately 1:1.33. Among traumatic cases, patients presented with various diagnoses, distributed as follows: extradural hematoma was observed in 110 patients, making up 34.27% of traumatic cases and 19.57% of all cases. Acute subdural hematoma was diagnosed in 94 patients, representing 29.28% of traumatic cases and 16.73% of the total. Hemorrhagic contusion was found in 34 patients, accounting for 10.59% of traumatic cases and 6.05% overall. Depressed fracture was noted in 49 patients, comprising 15.26% of traumatic cases and 8.72% of the total, while open wound was identified in 21 patients, representing 6.54% of traumatic cases and 3.74% overall. Lastly, diffuse axonal injury was diagnosed in 13 patients, making up 4.05% of traumatic cases and 2.31% of the total (Table 1).

**Table 1:** Distribution of diagnoses in traumatic neurosurgical emergency cases

Diagnosis	Frequency (% of traumatic cases, n = 241)	Frequency (% of total cases, n = 562)
Extradural hematoma	110 (34.27%)	110 (19.57%)
Acute subdural hematoma	94 (29.28%)	94 (16.73%)
Hemorrhagic contusion	34 (10.59%)	34 (6.05%)
Depressed fracture	49 (15.26%)	49 (8.72%)
Open wound	21 (6.54%)	21 (3.74%)
Diffuse axonal injury	13 (4.05%)	13 (2.31%)
Total	241 (100.00%)	241 (42.88%)

Among nontraumatic cases, patients presented with a range of diagnoses. Non-tumor hydrocephalus was the most common, affecting 70 patients (29.05% of nontraumatic cases; 12.46% of total cases). Brain tumors requiring CSF diversion, but not located in the posterior fossa, were diagnosed in 35 patients (14.52% of nontraumatic cases; 6.23% of total cases). Posterior fossa tumors necessitating CSF diversion were identified in 18 patients (7.47% of nontraumatic cases; 3.20% of total cases). Shunt malfunction occurred in 23 patients (9.54% of nontraumatic cases; 4.09% of total cases).

Intracerebral hematoma and chronic subdural hematoma were each diagnosed in 37 patients, representing 15.35% of nontraumatic cases and 6.58% of total cases. Postoperative tumor hematoma was observed in four patients (1.66% of nontraumatic cases; 0.71% of total cases), while postoperative vascular surgery hematoma was identified in one patient (0.41% of nontraumatic cases; 0.18% of total cases).

Infectious conditions were also noted, with infected wounds found in eight patients (3.32% of nontraumatic cases; 1.42% of total cases) and brain abscesses in six patients (2.49% of nontraumatic cases; 1.07% of total cases). Additionally, subdural hygroma was diagnosed in two patients (0.83% of nontraumatic cases; 0.36% of total cases) (Table 2).

**Table 2:** Distribution of diagnoses in non-traumatic neurosurgical emergency cases

Diagnosis	Frequency (% of non-traumatic cases, n = 321)	Frequency (% of total cases, n = 562)
Non-tumor hydrocephalus	70 (29.05%)	70 (12.46%)
Brain tumors (non-posterior fossa, requiring CSF diversion)	35 (14.52%)	35 (6.23%)
Posterior fossa tumor (requiring CSF diversion)	18 (7.47%)	18 (3.20%)
Shunt malfunction	23 (9.54%)	23 (4.09%)
Intracerebral hematoma	37 (15.35%)	37 (6.58%)
Chronic subdural hematoma	37 (15.35%)	37 (6.58%)
Postoperative tumor hematoma	4 (1.66%)	4 (0.71%)
Postoperative vascular surgery hematoma	1 (0.41%)	1 (0.18%)
Infected wound	8 (3.32%)	8 (1.42%)
Brain abscess	6 (2.49%)	6 (1.07%)
Subdural hygroma	2 (0.83%)	2 (0.36%)
Total	321 (100.00%)	321 (57.12%)

During the study period, a total of 562 surgical procedures were performed in the emergency operation theater, distributed as follows: craniotomy and evacuation of extradural hematoma were performed in 106 patients, accounting for 18.86% of the procedures. Craniotomy/craniectomy and evacuation of acute subdural hematoma were carried out in 75 patients, representing 13.35% of the procedures. Craniotomy and evacuation of spontaneous intracerebral hematoma were performed in 29 patients, comprising 5.16% of the procedures, while craniotomy and evacuation of hemorrhagic contusion were done in 27 patients, accounting for 4.80% of the procedures. Burr hole drainage was performed in six patients (1.07%), and burr hole evacuation was conducted in 39 patients (6.94%). Elevation of depressed fracture was carried out in 47 patients (8.36%), and wound debridement for open fractures and infected wounds was performed in 29 patients (5.16%). Ventriculoperitoneal shunt placement was carried out in 79 patients (14.06%), while external ventricular drain placement was performed in 39 patients (6.94%), and shunt revision in 23 patients (4.09%). Revision surgery for hematoma evacuation was conducted in nine patients (1.60%), and tracheostomy was performed in 54 patients (9.61%) (Table 3).

**Table 3:** Distribution of surgical procedures in the emergency operation theater

Procedure	n	%
Craniotomy and evacuation of extradural hematoma	106	18.86%
Craniotomy/craniectomy and evacuation of acute subdural hematoma	75	13.35%
Craniotomy and evacuation of spontaneous intracerebral hematoma	29	5.16%
Craniotomy and evacuation of hemorrhagic contusion	27	4.80%
Ventriculoperitoneal shunt placement	79	14.06%
External ventricular drain placement	39	6.94%
Elevation of depressed fracture	47	8.36%
Burr hole evacuation	39	6.94%
Wound debridement	29	5.16%
Tracheostomy	54	9.61%
Shunt revision	23	4.09%
Revision surgery for hematoma evacuation	9	1.60%
Burr hole drainage	6	1.07%
Total	562	100.00%

## Discussion

The audit conducted at the PINS, Lahore, Pakistan, sheds light on the significant burden of emergency cranial neurosurgery, with 562 cases managed over six months at this leading specialized center. Among the cases, 241 (42.88%) were traumatic, primarily involving extradural hematoma in 110 patients (34.27% of traumatic cases; 19.57% of total cases), acute subdural hematoma in 94 patients (29.28% of traumatic cases; 16.73% of total cases), and hemorrhagic contusion in 34 patients (10.59% of traumatic cases; 6.05% of total cases). Nontraumatic cases accounted for 321 (57.12%) of the total, with prominent conditions such as non-tumor hydrocephalus in 70 patients (29.05% of nontraumatic cases; 12.46% of total cases), intracerebral hematoma in 37 patients (15.35% of nontraumatic cases; 6.58% of total cases), and shunt malfunction in 23 patients (9.54% of nontraumatic cases; 4.09% of total cases). These findings align with the broader literature. Ullah et al. identified road traffic accidents as the leading cause of TBI in Pakistan, a trend likely reflected in the 241 trauma cases in this audit, consistent with the country’s high vehicular injury rates [15]. Clark et al. provided global estimates, noting that approximately 27 million annual TBI cases exist worldwide, with 4% (about 1.08 million) requiring neurosurgery. This global context frames the relevance of our frequent craniotomies, including 106 cases (18.86%) for extradural hematoma [16]. The higher proportion of nontraumatic cases, 321 (57.12%), exceeding that of trauma, is atypical for low- and middle-income countries and may be due to the Institute’s role as a referral hub for complex cases like hydrocephalus, as well as potential gaps in regional primary neurosurgical care. This observation warrants further investigation into regional care distribution.

A notable gender disparity emerged, with males comprising 415 (73.84%) of the cases, reflecting global trends of increased traumatic injury risk among men. Theodosopoulos et al. reported a male majority of 61% in neurosurgical procedures, attributing this to behavioral and occupational factors. They suggested gender-specific prevention strategies, such as safety campaigns targeting male-dominated activities like driving or manual labor [17]. Additionally, Obande et al. reported that 71% of neurosurgical ICU deaths in Nigeria involved young males with head trauma, with all fatalities linked to delayed presentation. This suggests that late arrivals - potentially a common issue in Pakistan’s resource-limited settings - could exacerbate outcomes [18]. This gender disparity, combined with delays in access to care, underscores the need for targeted preventive measures and improved pre-hospital systems to reduce trauma burdens among men and enhance survival chances.

The range of surgical interventions performed was diverse. Craniotomy for extradural hematoma was carried out in 106 patients (18.86%), ventriculoperitoneal shunt placement in 79 patients (14.06%), and tracheostomy in 54 patients (9.61%), highlighting the critical role of these procedures in preventing mortality and long-term disability. Chelvarajah et al. identified systemic challenges such as bed shortages and operative delays in neurosurgical care, which are likely intensified at our center, given its two-theater capacity against a substantial caseload of 562 patients [19]. Attebery et al. documented a 27% mortality rate among 51 Tanzanian neurosurgical patients, where procedures like shunts and craniotomies were successfully performed despite basic infrastructure, suggesting that Pakistan could optimize its existing resources for better outcomes [20]. Furthermore, Schipmann et al., in reviewing 34 studies, critiqued broad quality indicators such as 30-day readmission rates, calling for disease-specific measures tailored to conditions like trauma and nontrauma cases. This indicates that while our procedural volume is robust, efficiency and outcome tracking could be improved with more refined systems [21].

The study’s findings carry significant implications for healthcare policy and planning in Pakistan. The high burden of neurosurgical emergencies underscores the urgent need for additional dedicated neurosurgical facilities to meet the growing demand. Addressing infrastructure gaps, enhancing trauma care systems, and implementing preventive measures are crucial steps to reduce the burden of neurosurgical emergencies and improve patient outcomes.

Limitations

The findings of this study, derived from a single neurosurgical center, may not be generalizable to the broader national context, highlighting the need for multicenter audits to gain a more comprehensive understanding of neurosurgical emergency patterns. The retrospective design introduces potential limitations, such as missing data or biases, despite efforts to ensure data quality. Additionally, data collected exclusively from hospital records may not capture all relevant information, such as complications or mortality rates, thus limiting the assessment of the overall effectiveness of neurosurgical services.

## Conclusions

This audit at the PINS, Lahore, analyzed 562 cranial neurosurgical emergencies over six months, revealing a near-equal distribution between traumatic (241 patients, 42.88%) and nontraumatic (321 patients, 57.12%) cases. The leading traumatic diagnoses were extradural hematoma (110 patients, 19.57%) and acute subdural hematoma (94 patients, 16.73%), while nontraumatic cases were dominated by non-tumor hydrocephalus (70 patients, 12.46%) and intracerebral hematoma (37 patients, 6.58%). Notable procedures included craniotomy (106 patients, 18.86%) and ventriculoperitoneal shunt placement (79 patients, 14.06), underscoring the center’s diverse procedural demands.

These findings underscore the critical need for additional neurosurgical facilities in Pakistan to ensure timely access to care and alleviate the strain on existing centers. Furthermore, preventive strategies aimed at reducing road traffic accidents, falls, and other causes of head injury could significantly reduce the incidence of traumatic neurosurgical emergencies. The study also calls for further research into the long-term outcomes and socioeconomic impacts of these emergencies, which will be crucial for shaping public health policies and providing better support for affected individuals and their families.
